# Quantitative Detection of Viable but Nonculturable *Vibrio parahaemolyticus* in Frozen Bivalve Molluscs

**DOI:** 10.3390/foods12122373

**Published:** 2023-06-15

**Authors:** Eleonora Di Salvo, Felice Panebianco, Antonio Panebianco, Graziella Ziino

**Affiliations:** 1 Department of Veterinary Sciences, University of Messina, Polo Universitario dell’Annunziata, Viale Palatucci snc, 98168 Messina, Italy; 2 Department of Veterinary Sciences, University of Turin, Largo Braccini 2, Grugliasco, 10095 Turin, Italy

**Keywords:** *Vibrio parahaemolyticus*, frozen seafood, food safety, viable but nonculturable, PMA-qPCR

## Abstract

*Vibrio parahaemolyticus* is a foodborne pathogen diffusely distributed in the marine environment and often isolated from raw seafood belonging to different species, mostly shellfish. Ingestion of under- or uncooked seafood contaminated by *V. parahaemolyticus* can cause severe gastrointestinal symptoms in humans. Due to its ability to withstand low temperatures, *Vibrio* spp. could survive in frozen seafoods for long periods by entering the viable but nonculturable state (VBNC) and may constitute an unrecognized source of food contamination and infection. In the present study, seventy-seven frozen bivalve molluscs (35 mussels; 42 clams) were subjected to the detection and enumeration of viable *V. parahaemolyticus* using standard culture methods. VBNC forms were detected and quantified by applying an optimized protocol based on Propidium Monoazide (PMA) and Quantitative PCR (qPCR). All samples were negative for both the detection and enumeration of *V. parahaemolyticus* by the standard culture methods. VBNC forms were detected in 11.7% of the samples (9/77), with values ranging from 1.67 to 2.29 Log CFU/g. Only clam samples were positive for the detection of VBNC forms. The results of this study highlighted that VBNC *V. parahaemolyticus* may be present in frozen bivalve molluscs. Further data on the prevalence of VBNC *V. parahaemolyticus* in frozen seafood are needed in order to perform a robust risk assessment.

## 1. Introduction

*Vibrio parahaemolyticus* is a Gram-negative foodborne pathogen naturally present in marine and estuarine environments throughout the world. As a typical pathogen in seafood, it is one of the safety issues concerning these products, and food poisoning outbreaks caused by *Vibrio* spp. have been reported worldwide [1,2,3]. This bacterium can be commonly isolated from raw seafood, especially shellfish, with 100-fold greater levels in filter-feeding shellfish than in the surrounding water. To bring the amounts of undesirable microorganisms to acceptable levels for human consumption, purification of bivalves in controlled waters is used. However, it has been reported that some *Vibrio* species are resistant to depuration and can persist and replicate in bivalve tissues [4,5]. The ingestion of raw, undercooked, or contaminated products is considered the main cause of human infection. The disease can result in diarrhea, headache, vomiting, nausea, abdominal cramps, low fever and, in some cases, septicemia, shock, coma, and even death [6,7]. Less commonly, this organism can cause skin infections when an open wound is exposed to warm seawater [8]. In contrast to The United States and Asia, *V. parahaemolyticus* infections are rarely reported in Europe [9]. However, occasional outbreaks have been reported in some Mediterranean countries and surveys suggest that the incidence of *V. parahaemolyticus* infections also seems to be on the rise in Europe [10,11,12,13]. In the European Union, of 326 food samples from Bulgaria, the Netherlands, and Sweden, 32 (9.8%) were positive for *Vibrio* spp. These positive results involved raw fish, shrimps, and lobsters from border inspection activities in the three countries. In 2019, four *Vibrio* outbreaks were reported by France and Italy and *V. parahaemolyticus* was identified as the agent in all French outbreaks [14]. According to the current European Legislation (EC Reg. No. 2073/2005 and amendments) [15], the evaluation of shellfish safety is based on *Escherichia coli* and *Salmonella* spp. as indicators of faecal pollution. However, several studies [16,17] have shown that faecal indicators represent an inappropriate marker of microbiological safety and are scarcely correlated with the presence of microorganisms typical of the aquatic environment, including *Vibrio* spp., and other pathogens found in small quantities (Norovirus, *E. coli* O157:H7). Therefore, in the EC Reg. No. 2073/2005, no specific criteria for the detection of pathogenic *V. parahaemolyticus* in seafood are foreseen but, considering the epidemiological significance of this foodborne pathogen, there is an essential need to also detect the bacterium with molecular biological techniques [18].

Frozen storage is a method commonly used by the food industry to maintain product quality by inhibiting microbial growth. It has been reported to be effective in achieving certain reduction rates of *V. parahaemolyticus* in fish fillets, crabs, octopuses, shrimp, and oyster meat [19,20,21,22,23,24,25]. However, *Vibrio* spp. has been detected in frozen seafood products [26,27,28,29,30]. In this regard, the survival of vibrios at low temperatures is affected by several factors, namely temperature, salinity [31], acidity [32], organic nutrients [33], etc. Chitin, some amino acids, peptides, or phosphates have shown protective effects for *V. cholerae* [34] and *V. parahaemolyticus* [25,35]. Furthermore, as described by Wong et al. [25], psychrotrophic strains may have better survival potential than other mesophilic strains in frozen products and could probably enhance the risk of vibrios in frozen foods. Temperatures of −10 °C could be more effective at inactivating *V. parahaemolyticus* than −20 or −30 °C. Liu et al. [36] observed that this bacterium was reduced, in raw oysters, by 4.55, 4.13, and 2.53 log MPN/g after six months of storage at temperatures of −10, −20, and −30 °C, respectively. In a similar study, Shen et al. [22] demonstrated a reduction in the bacterial population from an initial level of 8.59 log CFU/mL to 2.04 and 3.84 log CFU/mL after 15 days of storage at −18 and −30 °C, respectively.

During the breeding, processing, and storage of seafood, *V. parahaemolyticus* can enter a viable but nonculturable (VBNC) state due to the low temperatures or excessive salinity commonly used for food preservation [37,38]. In the VBNC state, microorganisms become undetectable by routinary control tests since they do not form colonies on standard culture media [39,40]. It has been suggested that the VBNC state is an adaptive strategy for the long-term survival of bacteria under unfavourable environmental conditions [40,41] and represents a serious risk to human health. Several types of stressful conditions may induce the VBNC state in foodborne pathogens, including freezing, refrigeration, cooking, fermentation, and additive addition [42]. In addition, some food processing techniques, including high-pressure processing, electrolyzed water, pulsed electric field, pulsed light, nonthermal plasma, irradiation, ozone, and thermosonication [43,44,45] may also induce bacteria into the VBNC state [46]. To date, more than 100 bacterial and fungal species, most of which are pathogenic, have been found to enter the VBNC state in several foods, such as milk, dairy products, meat and meat products, seafoods, fruits and vegetables, juices, wine, and beer [47,48]. This list also includes many marine bacteria, including *Vibrio* species [49,50]. VBNC cells show important morphological changes, such as a reduction in cell size and/or modifications in membrane composition and the cell wall [41]. Additionally, survival ability under harsh environmental stresses, and physiological and molecular differences between VBNC and cultivable cells have been reported, such as RNA amount, gene expression, profiles of proteins, ATP synthesis, virulence, metabolism, and physical and/or chemical resistance [48]. Although the pathogenicity of VBNC cells per se is controversial, there is irrefutable evidence that they can regain infectivity and pathogenicity after resuscitation, causing human illness or food spoilage [40,51,52,53]. This resuscitation from the VBNC state requires specific conditions, including the removal of stressors, addition of rich nutrients, osmotic pressure stabilisation, hydrogen peroxide degradation, and host presence [54,55,56]. In particular, it was found that a temperature upshift may be strongly associated with the reversibility of VBNC bacteria [57,58]. VBNC bacteria in food environments may therefore represent a serious risk for human health as a result of potential underestimations of the total viable cells. In addition, their ability to resuscitate also endangers human life. For these reasons, in recent years, researchers have developed several culture-independent techniques based on the distinct characteristics of VBNC microbial cells, but each of them has pitfalls and limitations [47].

Molecular techniques, such as real-time polymerase chain reaction (qPCR), loop-mediated isothermal DNA amplification (qLAMP) for target gene amplification, and fluorescent in situ hybridization (FISH) can be used as alternatives to conventional methods for detecting, identifying, and quantifying microorganisms [59,60,61]. However, qPCR fails to distinguish among non-viable and viable cells, which can result in false positives [47,62]. To surmount this limitation, nucleic acid intercalating dyes, such as propidium monoazide (PMA) and ethidium monoazide (EMA), were applied to qPCR to discriminate and quantify VBNC microorganisms [23,37,63,64,65,66,67]. These nucleic acid dyes, in fact, penetrate and covalently bind to genomic DNA after photoactivation in non-viable cells, reducing the amplification of stained DNAs from dead cells [58]. PMA is indicated as a better dye, since EMA is known to bind also to the DNA of viable cells [68,69,70]. As described by several authors [68,70,71], PMA-qPCR is the preferred method for distinguishing between viable from non-viable cells. Hence, it may be used as a method for the quantitative detection of bacteria in the VBNC state [72]. This method was used for the detection of various VBNC bacteria cells, such as *Escherichia coli* O157:H7, *Campylobacter jejuni*, *V. cholerae*, *V. parahaemolyticus*, and other bacteria [23,37,56,64,66,73,74].

The detection of live bacteria in food is crucial not only for food safety concerns, but also to avoid useless product recalls and economic losses [75]. Furthermore, the detection of pathogenic bacteria, potentially occurring even in the VBNC state, in food preserved at low temperatures could provide important information for food contamination risk assessments. Even if the presence of VBNC bacteria in food is well documented [76,77,78], data on the occurrence of *V. parahaemolyticus* in frozen bivalve molluscs are still extremely limited. The aim of this study was to detect and quantify VBNC *V. parahaemolyticus* in frozen bivalve molluscs regularly marketed in Italy by means of species-specific PMA-qPCR and plate count techniques.

## 2. Materials and Methods

### 2.1. Samples Preparation

The present investigation was carried out on 77 samples of frozen bivalve molluscs, including clams (n. 42/77; 54.5%) and mussels (n. 35/77; 45.5%), purchased from different stores of mass-market retailers in Sicily (Italy) from February 2020 to January 2021. All samples were sold in plastic trays, individually sanitized, weighing between 200 and 500 g, and kept at a temperature of −25 ± 1 °C. From the label, it was possible to trace the FAO fishing area of each sample and the shelf life from a minimum of 18 to a maximum of 24 months. All samples were taken before the expiration date (Table 1).

Clam samples included n. 35/42 (83.3%) shelled and n. 7/42 (16.7%) whole-shell products belonging to five species [*Paphia undulata* (n. 13/42; 31.0%), *Chamelea gallina* (n. 13/42; 31.0%), *Paphia textile* (n. 10/42; 23,8%), *Meretrix lyrata* (n. 4/42; 9.5%), *Meretrix meretrix* (n. 2/42; 4.8%)] and three fishing areas [n. 25/42 (59.5%) from FAO zone 71, n. 2/42 (4.8%) from FAO zone 37, n. 15/42 (35.7%) from FAO zone 61]. Mussels included n. 32/35 (91.4%) shelled, n. 2/35 (5.7%) half-shell, and n. 1/35 (2.9%) whole-shell products all belonging to one species (*Mytilus chilensis*) and from the same fishing area (FAO zone 87). Samples were transferred to the laboratory under cold conditions and analysed within 24 h. One hundred grams of each sample were first thawed (<5 °C for 4 h) to avoid heat shock, then treated in a water bath at <20 °C for 15–20 min, homogenized, and divided into aliquots of 25 g. Each aliquot was diluted with sterile alkaline saline peptone water (ASPW) (pH 8.6 ± 0.2; Biolife, Milan, Italy) in a ratio of 1:9 (*w*/*v*) and homogenized through a stomacher (Stomacher^®^ 400 Circulator, International PBI s.p.a., Milan, Italy) for 2 min. Samples thus prepared were processed to determine *V. parahaemolyticus* via standard culture methods and PMA q-PCR to discriminate VBNC cells.

### 2.2. Detection and Enumeration of V. parahaemolyticus via Standard Culture Methods

The enumeration of *V. parahaemolyticus* was performed in duplicate on Thiosulfate Citrate Bile Sucrose (TCBS) (Difco, Le Point de Claix, France) agar plates, incubated at 37 ± 1 °C for 24 h, according to the EN ISO 21872-1:2017 [79]. For the detection, a pre-enrichment was instead performed in ASPW (Biolife), incubated at 37 ± 1 °C for 24 h. Subsequently, a loopful was spread on TCBS (Difco) agar plates, and, after incubation, plates were checked for the presence/absence of typical colonies.

### 2.3. PMA Assay and Genomic DNA Extraction

The PMA-qPCR assay was performed for the detection of VBNC *V. parahaemolyticus* as described by Liu et al. [64] with slight modifications. PMA dye (Biotium, Inc., Hayward, CA, USA) was dissolved in high-purity water to obtain a 20 mM stock solution and stored at −20 °C in the dark, according to the manufacturer’s instructions. Samples were then divided into two groups: the control and the PMA treated group. PMA was added to 500 μL live and heat-inactivated culture aliquots to a final concentration of 0, 10, 15, 20, 50, and 100 μM in the dark for 5, 10, and 15 min. A prolonged incubation period also favoured the decrease of the PCR signal from the heat-killed cells [56].

Considering the CT values for the viable and killed-cell aliquots (~10^9^ CFU/mL), we determined that 20 μM of PMA for 10 min was a consistent value for discrimination, and, consequently, it was chosen as the treatment value for the samples. At the end of the incubation, samples were exposed to light from a 650 W halogen light source at 20 cm on ice for 30 min. After photoinduced cross-linking, the free PMA was removed by centrifugation at 5000 rpm for 10 min before DNA extraction. DNA was isolated from 200 μL of the suspensions, PMA-treated or untreated, using NucliSENS easyMag unit (BioMérieux, Marcy-l’Étoile, France) to a final 20 μL extraction following the manufacturer’s protocol. The quality and concentration of DNA extracts were assessed at 260/280 and 260/230 nm using a SmartSpec Plus spectrophotometer (Bio-Rad, Milan, Italy). All DNA preparations were stored at −20 °C until use.

### 2.4. Real-Time qPCR Analysis

All frozen bivalve mollusc samples were examined by qPCR and PMA-qPCR. The target gene in this study was the *tlh* gene of *V. parahaemolyticus*, and the primer sequences were designed according to Bej et al. [80] as follows: forward primer Ltl 5′-AAAGCGGATTATGCAGAAGCACTG-3′ and reverse primer Rtl 5′-GCTACTTTCTAGCATTTTCTCTGC-3′. The length of the amplicon was 450 bp. The specificity of primers was assessed employing 17 bacterial strains (Table 2). PCR amplification mixture contained 10 μL of PowerUp SYBR Green Master Mix (Applied Biosystems, Monza, Italy), 2 μL of template DNA, 2 μL of each primer (final concentration 0.6 µM), and 4 μL nuclease-free water in a final volume of 20 μL. The cycling parameters were: 2 min at 50 °C, 2 min at 95 °C followed by 40 cycles of 15 s at 95 °C and 1 min at 60 °C. The qPCR was performed using the CFX96 TouchTM (Bio-Rad) with the Bio-Rad CFX manager 3.0 software.

### 2.5. Bacterial Strain and Growth Conditions

The reference strain of *V. parahaemolyticus* ATCC 17802, provided in freeze-dried form, was preserved in 20% (*v*/*v*) glycerol broth at −80 °C, then grown on nutrient agar (NA) (Biolife) supplemented with 3% (*w*/*v*) NaCl. After incubation at 37 °C for 18–24 h, a loopful of a colony was picked up and subsequently suspended in tryptic soy broth (TSB; Biolife) supplemented with 3.0% (*w*/*v*) NaCl, incubated with shaking (200 r/min) at 37 °C overnight to make cell suspensions. *V. parahaemolyticus* cells from log phase (OD600 = 1.0) were harvested by centrifugation at 8400 rpm for 5 min at 4 °C and then washed twice with an equal volume of artificial sea water (ASW) to make cell suspensions. ASW was prepared with sea salt (Sigma Aldrich, Milan, Italy) at a concentration of 30 g/L and it was filter-sterilized using 0.22 μm membrane filters (Merck, Darmstadt, Germany).

### 2.6. Preparation of Heat-Killed Cell Suspension

The heat-killed cell suspension was prepared by treating the cell suspension at 90 °C for 15 min. The absence of culturable cells was confirmed by pour-planting on 3% NaCl tryptic soy agar (TSA, Biolife) and the absence of any colony formation after incubation at 37 °C for 24 h.

### 2.7. Standard Curve

**Figure 1:**
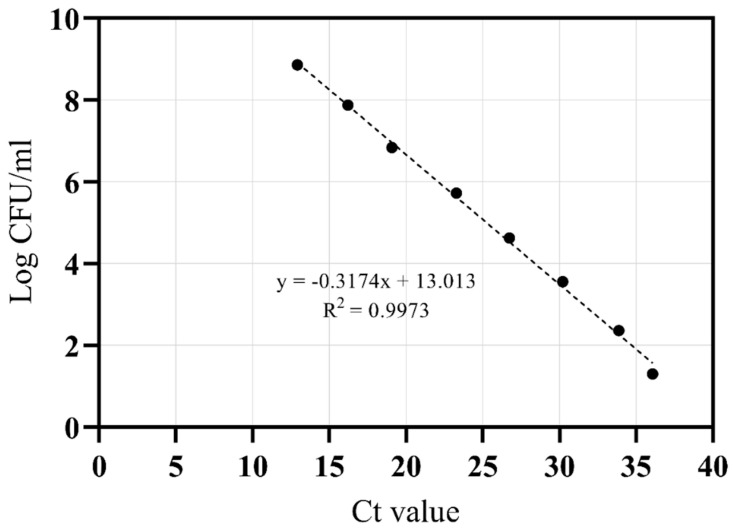
Mean standard curve obtained for *V. parahaemolyticus* ATCC 17802.

For the standard curves (Ct values versus log10 CFU/mL), 10-fold serial dilutions starting with 10^9^ CFU/mL of overnight cultures of *V. parahaemolyticus* ATCC 17802 were prepared, and bacteria were enumerated by the classic plate-counting method ranging from 10^9^ to 10^1^ CFU/mL. One millilitre of each dilution was treated with PMA under optimal conditions, the DNA template was extracted, and then the specific target was amplified in three experiments. The signals (Ct) were plotted against the log10 CFU/mL. Correlation coefficients (R2) and the amplification efficiencies were obtained as described by Rasmussen [81]. The limit of detection (LOD), and, consequently, the sensitivity of the PMA RTqPCR, was verified from the serial dilution of each standard prepared according to Caraguel et al. [82]. There was a strong linear correlation (r^2^ = 0.997) between the Ct value and log10 CFU/mL in the range. The detection limit was 1.30 log CFU/mL. In this study, results with Ct values greater than 36 were considered negative (Figure 1).

### 2.8. Statistical Analysis

Data of positive samples were analysed with a two-way ANOVA followed by a Tukey’s multiple comparison test (*p* < 0.05; GraphPad Prism version 9.0.0, GraphPad Software, San Diego, CA, USA) to detect significant differences among the samples in terms of CT values and predicted Log CFU/g.

## 3. Results

All the analysed samples were negative both for the detection and enumeration of *V. parahaemolyticus* by the standard culture methods (see Section 2.2). The qPCR and PMA-qPCR analyses resulted in the detection of putative VBNC forms only for clam samples. Fourteen samples (18.2% of all samples) were, in fact, positive in the qPCR detection. PMA-qPCR was able to discriminate between dead bacteria and VBNC forms, confirming the presence of VBNC *V. parahaemolyticus* in nine (11.7% of all samples) of the 14 qPCR-positive samples with predicted Log values ranging from 1.67 to 2.29 Log CFU/g (Table 3). 

The nine VBNC positive samples belonged to the category of shelled clams and included three species [*Chamelea gallina* (n. 4/9; 44.4%), *Paphia undulata* (n. 4/9; 44.4%), *Paphia textile* (n. 1/9; 11.1%)] and two fishing areas [n. 8/9 (88.9%) from FAO zone 71, n. 1/9 (11.1%) from FAO zone 61].

## 4. Discussion

In recent years, researchers have focused on the study of VBNC forms due to the wide variety of foodborne pathogens that can enter this state. VBNC cells, in fact, could pose a serious threat to food safety, given their ability to escape from traditional detection and enumeration methods, to withstand harsh situations (heat treatment, antibiotics, etc.), and to resuscitate and potentially lead to disease in consumers [39,40,46]. Therefore, the advancement of quick and efficient approaches for the detection of VBNC bacteria in food has become an urgent need. PMA-qPCR has been proven effective in differentiating live from dead bacteria [23,37,56,58,64,66]. In fact, different protocols have been developed to selectively identify VBNC *V. parahaemolyticus* cells employing PMA associated with several molecular methods [23,37,64,65,66,67]. Furthermore, different genetic markers were employed in PCR assays for detection of *V. parahaemolyticus,* including *tdh, trh, toxR*, 16s rDNA, *pR72H, gyrB,* and *vp1332* [83,84,85,86,87]. The thermolabile haemolysin gene (*tlh*) encoding a phospholipase A2 is considered a species-specific marker for *V. parahaemolyticus* and is often utilised to identify this species [80,88]. Therefore, the unique fragment of the *tlh* gene was selected as the genetic marker in this study. The sensitivity of the PMA-qPCR assay was tested using serial dilutions of *V. parahaemolyticus* genomic DNA template. The LOD of PMA-qPCR was calculated to be 1.30 log CFU/mL, with the benefit of rapidity and specificity versus the standard culture method. Other qPCR assays developed in previous studies reported LODs for *V. parahemolyticus* of 1.2 × 10^2^ CFU/mL [64], 5 × 10^1^ CFU/g [63], 12 CFU/reaction [66], and 28 CFU/g [62]. Previous studies indicated that different factors, such as the quantification of all cells (dead and viable) compared to viable cells, the use of various dyes (e.g., PMA or EMA), bacteria, primers, protocols for DNA extraction, and the qPCR procedure could contribute to the variation of the reported LODs. In addition, the optimal concentration of PMA varied among the different studies. Furthermore, it is known that these methodologies must be validated for each matrix type to prevent an overestimation of VBNC cells resulting from the occurrence of dead cells with intact membranes [89]. As reported by several authors [68,76,89,90], higher PMA concentrations might interfere with the DNA amplification of viable cells, leading to underestimations in viable cell quantifications or erroneous results. Conversely, a lower PMA concentration might not be completely effective in inhibiting the signal from the dead cells, causing overevaluation. However, several studies have demonstrated that the capacity of PMA to eliminate the signal from dead-cell DNA increases sharply with increased PMA concentrations [56,64]. As reported by Fittipaldi et al. [68], the time that cells are exposed to a viability dye must be long enough to allow the chemical to enter membrane-compromised cells and intercalate into their DNA. Incubation times must be viewed in the context of the microbial species targeted and the concentration of the dye applied. By using low dye concentrations, incubation times can be considered more flexible. For bacteria, an incubation time of five minutes is generally accepted and more commonly used [68]. In our study, at 20 µM and incubation time of 10 minutes, PMA treatment eliminated more than 99% of the signal from the dead cell’s DNA compared with the untreated control. Recently, novel detection methods were developed for VBNC cells, including phage-mediated detection systems, biosensors, microfluidic-based techniques, D_2_O-labeled Raman spectroscopy, matrix-assisted-laser desorption ionization time-of-flight mass spectrometry (MALDI-TOF-MS), liquid chromatography–mass spectrometry, autoradiography, ATP generation, and DNase I protection assay [41,44]. However, for the moment, there is not much data on their applications in food products.

During the last decades, diseases triggered by foodborne pathogens have become a significant public health issue worldwide. Among these, *V. parahaemolyticus* was frequently responsible for causing disease, becoming a big threat for public safety [91]. In particular, the higher consumption of raw or uncooked seafood has increased interest in this microorganism. However, the ability of *V. parahaemolyticus* to tolerate low temperatures, and its detection in frozen products, extends the range of dangerous foods for consumers including also frozen seafood [23,92]. Several factors affect the viability of *V. parahaemolyticus* during freezing and thawing, such as the extracellular concentration of solutes in the food, growth phase of the microorganism, presence of natural cryoprotectants (chitin, some amino acids, or peptides), pH, temperature, freezing rate, exposure to chemical stresses before freezing, amount of nutrients in the environment, cooling or freezing medium composition, rate of cooling, dissolution, thawing speed, etc. [93]. Studies conducted on oyster meat, octopus, crabs, and fish fillets [20,24,25] show that, after an initial rapid decrease, *Vibrio* is able to adapt to low temperatures through modifications of the composition of the fatty acids of the cell membrane and protein synthesis [25], remaining viable and potentially pathogenic for a long time. In addition, like other bacteria, *Vibrio* spp. can enter the VBNC state during freezing. A recent study [23] showed that the risk of *V. parahaemolyticus* persisted in sea bass stored at 4 °C, −18 °C and −45 °C for 14 days; the fastest decrease in culturability, and a higher level of transition to the VBNC state were observed in samples stored at sub-zero temperatures. This is worrying, considering that studies have demonstrated how VBNC forms of *Vibrio* can become active again after cold storage and freezing due to temperature upshifts [94,95]. This could lead to a growth of *Vibrio* in products during thawing and, if the foods are not subsequently cooked adequately, cells that have regained their viable state could persist and cause disease. 

In this study, all samples were negative for viable *V. parahaemolyticus* with culture-dependent methods, while PMA-qPCR detected and quantified VBNC *V. parahaemolyticus* in 11.7% of culture-negative samples. Only clam samples were positive for VBNC *V. parahaemolyticus*. While there are numerous studies on the use of PMA-qPCR on artificially contaminated seafood samples, including shrimp, crab, fish, and shellfish [23,62,64,66,96], few of them have addressed the efficacy of these methods for detecting *V. parahaemolyticus* in marketed seafood. Yu et al. [96], in a comparative study using real-time fluorescent PMA-LAMP and PMA-qPCR on 139 fishery product samples, including cod, grilled croaker, dried squid, and shrimp, reported a positivity of 2.16% for both methods.

Niu et al. [97], in a study aimed at establishing a novel qPCR for the simultaneous detection and quantification of viable pathogenic and non-pathogenic strains of *V. parahaemolyticus* in raw shrimp (*Penaeus vannamei*) and clams (*Ruditapes philippinarum*) purchased from various regions of Shanghai, did not find *V. parahaemolyticus* in any sample. In a study conducted by Zhu et al. [66] to investigate the utility of the PMA-qPCR method, and quantify *tdh*-positive viable cells of *V. parahaemolyticus* in raw seafood, including oyster, scallop, shrimp, and crab, viable *V. parahaemolyticus* were isolated from three (10%) oyster samples, two (6.7%) shrimp samples, two (6.7%) crab samples, and one (3.3%) scallop sample. Recently, some studies on a new DNA-intercalating agent, PMAxx, produced on the basis of PMA, would have shown greater activity and better ability to distinguish between live and dead bacteria [98]. However, PMAxx-applied research on seafood is rarely reported at present [62,67,99]. 

Our results are substantially in accordance with other studies on viable *V. parahaemolyticus* in frozen products. In fact, viable *Vibrio* spp., including *V. parahaemolyticus*, are commonly isolated from fresh bivalve shellfish worldwide [100,101,102] but their occurrence in frozen products is less common. Panebianco et al. [28], on 81 samples of frozen bivalve molluscs, isolated *V. parahaemolyticus* in 3.24% of samples (clams, scallops, mussels). Tang et al. [27], in frozen molluscs marketed in Malaysia, reported higher percentages (43.75% with the classic method, and 57.5% with PCR), but the authors did not exclude the possibility of cross-contamination in the marketing phases for contact with other fishery products and ice. Similar results showing clams as the main source of *V. parahaemolyticus* were obtained by Lopatek et al. [103] (31 of 120 clam samples, 25.8%) and Roque et al. [104] (38 of 90 clam samples, 42.2%). Lamon et al. [100,102] detected higher loads of *Vibrio* spp. in clams (*Ruditapes decussatus*) than in mussels (*Mytilus galloprovincialis*) collected in Sardinia (Italy). According to these authors, the detection of *V. parahaemolyticus* only in clams in our study could be linked to the harvesting method. Clams, in fact, are usually harvested in muddy sand sediments while mussels are collected in ropes suspended in the water column. Since it has been demonstrated that loads of *Vibrio* spp. are higher in the sediments compared to the water column, the detection of VBNC *V. parahaemolyticus* only in clam samples in our study appears justifiable [42,100,102,105]. Another explanation for the positive detection only in clam samples may be related to the handling undergone by the products during the shelling process. Shen et al. [22] demonstrated that at −18 °C the populations of *V. parahaemolyticus* were reduced to non-detectable levels in shucked oysters after 60 days (<3.8 log MPN/g) and to 0.38 log MPN/g in shell stock oysters after 75 days. These data suggested that frozen storage is an effective method for reducing *V. parahaemolyticus*, but also that *Vibrio* levels were higher in shelled than in shucked oysters. We can hypothesize that a preliminary handling of molluscs during the shelling process could result in higher initial bacteria loads and, consequently, more VBNC forms during freezing. Another factor to consider is seasonality. In this regard, a study [106] showed that lab-grown *Vibrio* were eliminated by oysters after depuration, while naturally present *Vibrio* persisted. This did not explain, however, why oysters that initially seemed characterized by low levels of *Vibrio* suddenly contained several logs more of such bacteria after exposure to a different bacterial population. This may be the consequence of a natural bacterial population already existing in a viable but non culturable state during cold months. In addition, it may be possible that the bacterium enters this form to survive the decreased temperatures. Several studies reported the absence of viable *Vibrio* in oysters during cold-water months, and their re-appearance when warmer waters return [107,108,109]. The findings presented here offer further insights as to how and why cells present in molluscs in the VBNC state become detectable upon the increase of water temperatures and the bacterial communities of these waters during spring and summer months. 

The results of this study showed that VBNC forms of *V. parahaemolyticus* can persist in bivalve molluscs subjected to freezing and confirm that this bacterium may represent a concern for consumers due to its adaptation abilities. Although little information is available on the role of a VBNC state in foodborne outbreaks, due to limitations in the detection and traceability of the original source, it is not possible to exclude that VBNC bacteria could be implicated in foodborne diseases. For instance, an outbreak due to salted salmon roe containing VBNC *E. coli* O157:H7 was reported in Japan [110]. Asakura et al. [111] have instead suggested that *Salmonella Oranienburg* could enter the VBNC state after NaCl stress in the outbreak provoked by dried processed squids; this assumption was corroborated by resuscitation trials. In 2011, the possibility that an initially undetected *E. coli* O104:H4 strain was responsible for a large outbreak (about 3000 cases) causing enterohemorrhagic and enteroaggregative diseases was discussed by Aurass et al. [112]. Even if there is no proof that VBNC pathogens directly led to these outbreaks, the above-mentioned surveys emphasise how the possible presence of VBNC pathogens can pose a serious risk to public health. As reported by Nicolò and Guglielmino [113], 20% of illnesses can be associated with well-known pathogens, but the other 80% are caused by unspecified or unidentified agents, suggesting that pathogens in the VBNC state might be neglected during most outbreaks because of their non-detectability. Considering that cold and frozen storage are the most common methods applied for the preservation of seafood products, more data on the presence of VBNC forms of *Vibrio* in frozen products are needed. Additionally, studies about the presence of VBNC *Vibrio* in seafood that are preserved differently, such as in increasingly popular ethnic seafood [114], are required. In fact, *Vibrio* spp., including *V. parahaemolyticus*, were isolated from dried seafood [29,30,115] and it was demonstrated that VBNC forms of *V. parahaemolyticus* are resistant not only to cold temperatures but also to other stressors, such as heat, acidity, and low salinity [57]. 

## 5. Conclusions

The detection of VBNC bacteria appears crucial to avoid false-positive or false-negative results at the industrial food production level. Underestimating the product safety profile can lead to serious consequences, since the occurrence of pathogenic species in food can result in serious outbreaks, and the potential re-growth of VBNC microorganisms could lead to a reduction in shelf life. Although the presence of VBNC bacteria is well reported, to our knowledge, this is the first study to report the presence of VBNC *V. parahaemolyticus* in commercial frozen bivalve molluscs. The positive results detected only for clams are probably linked to several factors, such as the harvesting method, initial contamination of the product during shelling operations, and seasonality. Our results show that PMA-qPCR can be considered as a fast and reliable method for the detection of VBNC pathogenic bacteria in food or during food processing, representing a useful tool for a more realistic risk assessment of *V. parahaemolyticus* in seafood products. Further studies are needed to acquire data about the prevalence of viable but non culturable *V. parahaemolyticus* in frozen bivalve molluscs and other seafood stored by cold or other preserving methods that could facilitate the development of VBNC forms.

## Figures and Tables

**Table 1:** Characteristics and label information of frozen bivalve molluscs analysed in this study.

Sample ID	Species	Type	FAO Fishing Area	Sampling Date
1	*Paphia undulata*	shelled clams	71	10 February 2020
2	*Mytilus chilensis*	shelled mussels	87	10 February 2020
3	*Paphia undulata*	shelled clams	71	10 February 2020
4	*Mytilus chilensis*	half shell mussels	87	10 February 2020
5	*Mytilus chilensis*	shelled mussels	87	10 February 2020
6	*Chamelea gallina*	shelled clams	71	10 February 2020
7	*Mytilus chilensis*	shelled mussels	87	10 February 2020
8	*Paphia undulata*	shelled clams	71	10 February 2020
9	*Mytilus chilensis*	shelled mussels	87	16 March 2020
10	*Mytilus chilensis*	shelled mussels	87	16 March 2020
11	*Paphia undulata*	shelled clams	71	16 March 2020
12	*Mytilus chilensis*	shelled mussels	87	16 March 2020
13	*Paphia undulata*	whole shell clams	71	16 March 2020
14	*Chamelea gallina*	shelled clams	37	16 March 2020
15	*Mytilus chilensis*	shelled mussels	87	16 March 2020
16	*Chamelea gallina*	shelled clams	71	16 March 2020
17	*Chamelea gallina*	shelled clams	71	16 March 2020
18	*Mytilus chilensis*	shelled mussels	87	20 April 2020
19	*Chamelea gallina*	shelled clams	71	20 April 2020
20	*Paphia textile*	shelled clams	61	20 April 2020
21	*Chamelea gallina*	shelled clams	71	20 April 2020
22	*Paphia textile*	shelled clams	61	20 April 2020
23	*Chamelea gallina*	shelled clams	71	20 April 2020
24	*Mytilus chilensis*	shelled mussels	87	20 April 2020
25	*Chamelea gallina*	shelled clams	71	20 April 2020
26	*Mytilus chilensis*	shelled mussels	87	20 April 2020
27	*Mytilus chilensis*	shelled mussels	87	22 June 2020
28	*Mytilus chilensis*	shelled mussels	87	22 June 2020
29	*Meretix lyrata*	whole shell clams	61	22 June 2020
30	*Paphia undulata*	shelled clams	71	22 June 2020
31	*Mytilus chilensis*	whole shell mussels	87	22 June 2020
32	*Mytilus chilensis*	shelled mussels	87	22 June 2020
33	*Paphia undulata*	shelled clams	71	22 June 2020
34	*Mytilus chilensis*	shelled mussels	87	22 June 2020
35	*Mytilus chilensis*	shelled mussels	87	27 July 2020
36	*Paphia undulata*	shelled clams	71	27 July 2020
37	*Paphia textile*	whole shell clams	61	27 July 2020
38	*Mytilus chilensis*	shelled mussels	87	27 July 2020
39	*Paphia undulata*	shelled clams	71	27 July 2020
40	*Mytilus chilensis*	shelled mussels	87	27 July 2020
41	*Paphia textile*	shelled clams	37	27 July 2020
42	*Paphia textile*	shelled clams	61	27 July 2020
43	*Paphia textile*	shelled clams	61	27 July 2020
44	*Mytilus chilensis*	shelled mussels	87	7 September 2020
45	*Paphia undulata*	shelled clams	71	7 September 2020
46	*Mytilus chilensis*	shelled mussels	87	7 September 2020
47	*Chamelea gallina*	shelled clams	71	7 September 2020
48	*Mytilus chilensis*	shelled mussels	87	7 September 2020
49	*Mytilus chilensis*	shelled mussels	87	7 September 2020
50	*Meretrix meretrix*	whole shell clams	61	7 September 2020
51	*Paphia undulata*	shelled clams	71	7 September 2020
52	*Paphia undulata*	shelled clams	71	13 October 2020
53	*Mytilus chilensis*	whole shell mussels	87	13 October 2020
54	*Meretrix lyrata*	whole shell clams	61	13 October 2020
55	*Paphia undulata*	shelled clams	71	13 October 2020
56	*Mytilus chilensis*	shelled mussels	87	13 October 2020
57	*Paphia textile*	shelled clams	61	13 October 2020
58	*Mytilus chilensis*	shelled mussels	87	13 October 2020
59	*Meretrix lyrata*	shelled clams	61	13 October 2020
60	*Mytilus chilensis*	shelled mussels	87	13 October 2020
61	*Mytilus chilensis*	shelled mussels	87	14 December 2020
62	*Chamelea gallina*	shelled clams	71	14 December 2020
63	*Chamelea gallina*	shelled clams	71	14 December 2020
64	*Mytilus chilensis*	shelled mussels	87	14 December 2020
65	*Mytilus chilensis*	shelled mussels	87	14 December 2020
66	*Chamelea gallina*	shelled clams	71	14 December 2020
67	*Chamelea gallina*	shelled clams	71	14 December 2020
68	*Paphia textile*	shelled clams	61	14 December 2020
69	*Meretrix lyrata*	whole shell clams	61	11 January 2021
70	*Mytilus chilensis*	shelled mussels	87	11 January 2021
71	*Mytilus chilensis*	shelled mussels	87	11 January 2021
72	*Mytilus chilensis*	shelled mussels	87	11 January 2021
73	*Paphia textile*	shelled clams	61	11 January 2021
74	*Mytilus chilensis*	shelled mussels	87	11 January 2021
75	*Mytilus chilensis*	shelled mussels	87	11 January 2021
76	*Paphia textile*	shelled clams	61	11 January 2021
77	*Meretrix meretrix*	whole shell clams	61	11 January 2021

**Table 2:** Stains used in the present study and qPCR results.

No.	Species	Strain	Source	qPCR Result
1	*V. parahaemolyticus*	ATCC 17802	Shirasu food poisoning, Japan	+
2	*V. parahaemolyticus*	ATCC 33847	human clinical isolate	+
3	*V. parahaemolyticus*	CCUG 43363	unknown	+
4	*V. parahaemolyticus*	MELAB 772	mussels	+
5	*V. parahaemolyticus*	MELAB 777	mussels	+
6	*V. parahaemolyticus*	MELAB 778	mussels	+
7	*V. parahaemolyticus*	MELAB 547	fish	+
8	*V. alginolyticus*	ATCC 17749	fish	−
9	*V. vulnificus*	ATCC 27562	blood	−
10	*V. cholerae*	CCUG 37531	unknown	−
11	*V. mimicus*	CCUG 13624	human clinical isolate	−
12	*A. hydrophila*	ATCC 7966T	tin of milk with fishy odor	−
13	*A. molluscorum*	CECT 5864	wedge shells	−
14	*A. sobria*	CECT 4245T	fish	−
15	*E. coli*	ATCC 8739	feces	−
16	*L. monocytogenes*	ATCC 13932	human clinical isolate	−
17	*P. aeruginosa*	ATCC 15442	water	−

ATCC: American type culture collection. CCUG: Culture collection University of Gothenburg. MELAB: Food Microbiology Lab of Veterinary Sciences Department (Messina, Italy). CECT: Spanish Type Culture Collection.

**Table 3:** Samples positive to qPCR and PMA-qPCR and relative CT and Log CFU/g values.

Sample ID	Type	Species	FAO Fishing Area	Sampling Period	Plate Count	CT Values		Log CFU/g Predicted	
						qPCR (Dead + VBNC)	PMA-qPCR (VBNC)	qPRC (Dead + VBNC)	PMA-qPCR (VBNC)
6	shelled clams	*Chamelea gallina*	71	February 2020	UD	29.05 ^a^	UD	3.79 ^a^	UD
8	shelled clams	*Paphia undulata*	71	March 2020	UD	31.18 ^b^	34.54 ^a^	3.12 ^b^	2.05 ^a^
11	shelled clams	*Paphia undulata*	71	March 2020	UD	29.26 ^c^	35.1 ^b^	3.73 ^c^	1.87 ^b^
16	shelled clams	*Chamelea gallina*	71	March 2020	UD	32.08 ^d^	35.73 ^c^	2.83 ^d^	1.67 ^c^
19	shelled clams	*Chamelea gallina*	71	April 2020	UD	33.37 ^e^	34.74 ^d^	2.42 ^e^	1.99 ^d^
20	shelled clams	*Paphia textile*	61	April 2020	UD	30.21 ^f^	33.78 ^e^	3.42 ^f^	2.29 ^e^
23	shelled clams	*Chamelea gallina*	71	April 2020	UD	35.21 ^g^	UD	1.84 ^g^	UD
33	shelled clams	*Paphia undulata*	71	June 2020	UD	29.74 ^h^	35.58 ^f^	3.57 ^h^	1.72 ^f^
36	shelled clams	*Paphia undulata*	71	July 2020	UD	32.86 ^i^	UD	2.58 ^i^	UD
45	shelled clams	*Paphia undulata*	71	September 2020	UD	27.32 ^l^	34.62 ^g^	4.34 ^l^	2.03 ^a^
51	shelled clams	*Paphia undulata*	71	September 2020	UD	35.67 ^m^	UD	1.69 ^m^	UD
62	shelled clams	*Chamelea gallina*	71	December 2020	UD	35.59 ^n^	UD	1.72 ^n^	UD
63	shelled clams	*Chamelea gallina*	71	December 2020	UD	31.19 ^b^	35.51 ^h^	3.11 ^b^	1.74 ^f^
66	shelled clams	*Chamelea gallina*	71	December 2020	UD	33.65 ^o^	35.48 ^i^	2.33 ^o^	1.75 ^fg^

UD = Undetected. Values followed by different superscript letters in the same column are significantly different (ANOVA and Tukey’s multiple comparison; *p* < 0.05).

## Data Availability

The data used to support the findings of this study can be made available by the corresponding author upon request.
